# Measurement of Procalcitonin as an Indicator of Severity in Patients With Chronic Obstructive Pulmonary Disease Admitted With Respiratory Illness

**DOI:** 10.7759/cureus.28511

**Published:** 2022-08-28

**Authors:** Alexander J Davies, Paul W Blessing, Wesley P Eilbert

**Affiliations:** 1 Pulmonary and Critical Care Medicine, University of Maryland Medical Center, Baltimore, USA; 2 Emergency Medicine, University of Illinois at Chicago, Chicago, USA

**Keywords:** emergency department, pneumonia, copd exacerbation, risk stratification, procalcitonin

## Abstract

Introduction

Exacerbations of chronic obstructive pulmonary disease (COPD) are a frequent reason for hospital admission and a major cause of morbidity and mortality. A useful biomarker or indicator of disease severity at the time of presentation could help guide treatment and identify those with poor prognosis who need early aggressive intervention. We hypothesized that patients who present to the hospital with COPD exacerbations who are found to have elevated procalcitonin (PCT) levels will have worse outcomes such as longer admissions, increased intensive care unit (ICU) utilization, and more frequent readmissions than those with normal levels, regardless of presence or absence of infiltrate on initial chest X-ray (CXR).

Methods

We conducted a retrospective chart review of patients admitted to our facility with a respiratory complaint and a diagnosis of COPD to examine the relation between PCT and disease severity. A total of 156 unique encounters were reviewed, with 87 included in the final data set. Data was collected on baseline medical conditions as well as clinical status at the time of presentation. Primary endpoints included the need for overnight ICU admission, hospital length of stay greater than seven days, and repeat visit within 30 days of discharge. Secondary endpoints included the need for intubation at the time of admission, in-hospital mortality or discharge to hospice, and ICU length of stay.

Results

Patients with elevated PCT levels (>0.25ng/mL) had a significantly increased likelihood of a need for ICU admission (odds ratio 3.18) and hospital length of stay greater than seven days (odds ratio 3.38). There was no statistically significant difference in the Emergency Department readmission rate or any of the secondary outcomes.

Conclusions

Our data suggests that PCT may be a useful early biomarker for patients with COPD presenting with an acute respiratory illness.

## Introduction

Chronic obstructive pulmonary disease (COPD) is a condition that affects nearly one in five American adults over age 40 and exacerbations account for over half a million hospitalizations a year in the United States [1]. The disease is one of the leading causes of death in the USA with the total cost of COPD treatment in excess of 50 billion dollars a year [2]. It’s a well-established phenomenon that bacterial infections are part of the underlying trigger for many COPD exacerbations, and therefore antibiotics are a mainstay of treatment in a subset of patients [3]. Recent advances in laboratory testing for procalcitonin (PCT) have led to the widespread availability of PCT assays, and using these levels as a screening tool for a bacterial component of COPD exacerbations could lead to the decreased use of antibiotics [4,5].

The use of PCT to grade the severity of COPD exacerbations without regard to antibiotic use has only been investigated briefly. Pantzaris et al.’s 2018 review article found two studies that examined the relationship of PCT levels and the need for positive pressure ventilation [6-8]. They also identified one study that found a relationship between increased PCT levels and mortality in intubated patients with severe COPD exacerbations [9]. One retrospective study by Flattet et al. at an academic medical center in Switzerland performed multivariate analysis on a retrospective cohort of 129 COPD patients to attempt to determine the prognostic value of various tests and biomarkers. As part of their secondary analysis, they found a strong association with PCT level on admission for COPD exacerbation and all-cause mortality at five years [10]. Here, we attempt to evaluate the association of PCT levels with more immediate outcomes, including the need for intensive care unit (ICU) admission, hospital length of stay, and readmission rate.

## Materials and methods

Study design

We conducted a retrospective chart review of patients seen as inpatients or in the emergency department (ED) at our urban, academic, tertiary referral medical center during the 2017-2018 academic year. IRB approval (Protocol #2020-0005) was granted by the University of Illinois at Chicago where all data collection took place.

Study population

Patients with an admission or discharge diagnosis of COPD and at least one laboratory order for PCT level during their visit were included. Patients were identified with the assistance of the information services department, and BusinessObjects software was used for data extraction. The data set included patient information for visits between July 2017 to June 2018 with a J44 ICD-10 code (COPD) where at least one PCT level was ordered. This identified 137 unique patients with 156 total encounters (15 patients had two visits, two patients had three visits). Exclusion criteria included PCT level drawn during an outpatient visit only, patients in whom PCT level was tested for reasons unrelated to their pulmonary disease and patients admitted with a non-respiratory complaint (e.g., chest pain, sepsis), and the presence of end-stage renal disease (ESRD). Patients with ESRD were excluded specifically due to the unreliability of PCT in patients with renal impairment [11]. There were also 12 encounters excluded as a PCT level was ordered but canceled or not drawn.

Statistical analysis

At least 20 percent of identified charts were reviewed by multiple authors to ensure accuracy. Statistical analysis was performed with assistance from a research specialist within the department of emergency medicine. A PCT cutoff of 0.25ng/ml, the upper level of normal for our laboratory, was used to divide patients into low PCT and high PCT cohorts for the purpose of data analysis. P values were calculated using chi-squared analysis for bivariate associations, and odds ratios for rate comparisons.

Data collection and outcomes

Data was collected on age, gender, and baseline medical problems that may affect clinical outcomes, such as the presence of diabetes, HIV, and heart failure. The presence of lobar infiltrates on chest imaging, initial serum lactate level, and white blood cell count were recorded. Therapies received such as administration of corticosteroids and antibiotics were also recorded. Primary endpoints were a need for overnight ICU admission, hospital length of stay (LOS) greater than seven days, and repeat ED visit within 30 days of discharge. Secondary endpoints included a need for intubation at the time of admission, in-hospital mortality or discharge to hospice, and ICU length of stay.

## Results

Patient characteristics

**Figure 1:**
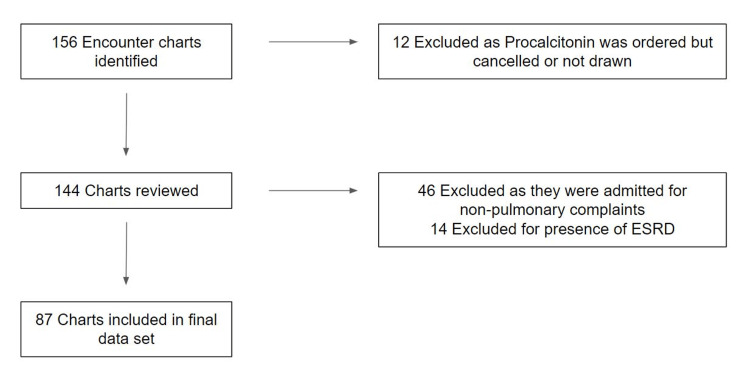
Identification of study population ESRD: end-stage renal disease

A total of 156 encounters were identified for evaluation. Eighty-seven encounters, 41 males and 46 females, met the final inclusion criteria (Figure 1). The median age was 64.2 years (range 37-94), and just over half (n=44, 50.6%) of the included patients had diabetes, cardiomyopathy with reduced ejection fraction, or HIV (Table 1). There was a significant correlation between an elevated PCT level and the presence of lobar consolidation (p=0.017), and the nine patients with markedly elevated serum lactate on arrival also had significantly increased PCT levels (6.39ng/mL vs 1.05ng/mL in the remainder of the cohort). There were more patients in the low PCT group that received corticosteroids on admission (77% vs 53%, p=0.022). The total number of patients with abnormal white blood cell counts as defined by >10x10⁹/L or <4x10⁹/L was not statistically different between high and low PCT groups (p=0.925).

**Table 1:** Baseline characteristics, presenting data, and initial treatments received * Denotes statistically significant difference between groups (p<0.05) DM: diabetes mellitus, CHF: congestive heart failure, HIV: human immunodeficiency virus, WBC: white blood cell count, <=: less than or equal to, > : greater than.

		All Patients	ProCalcitonin <=0.25ng/ml	ProCalcitonin >0.25ng/ml	P Value
Total	n	87	57 (65.5%)	30 (34.5%)	-
Age	>60 years	52 (59.8%)	37 (64.9%)	15 (50.0%)	0.178
	<=60 years	35 (41.2%)	20 (35.1%)	15 (50.0%)	
Gender	Female	46 (52.9%)	32 (56.1%)	14 (46.7%)	0.4
	Male	41 (47.1%)	25 (43.9%)	16 (53.3%)	
DM	Yes	31 (35.6%)	23 (40.3%)	8 (26.7%)	0.205
	No	56 (64.4%)	34 (59.7%)	22 (73.3%)	
CHF	Yes	14 (16.1%)	9 (15.8%)	5 (16.7%)	0.916
	No	73 (83.9%)	48 (84.2%)	25 (83.3%)	
HIV	Yes	4 (4.6%)	2 (3.5%)	2 (6.7%)	0.504
	No	83 (95.4%)	55 (96.5%)	28 (93.3%)	
Lobar Opacity	Yes	29 (33.3%)	14 (24.6%)	15 (50%)	*0.017
	No	58 (66.7%)	43 (75.4%)	15 (50%)	
WBC	>10 or <=4 cells x10^9^/liter	47 (54.0%)	31 (54.4%)	16 (53.3%)	0.925
	Normal	40 (66.0%)	26 (45.6%)	14 (46.7%)	
Received antibiotics	Yes	78 (89.7%)	50 (87.7%)	28 (93.3%)	0.414
	No	9 (10.3%)	7 (12.3%)	2 (6.7%)	
Received corticosteroids	Yes	60 (69.0%)	44 (77.2%)	16 (53.3%)	*0.022
	No	27 (31.0%)	13 (22.8%)	14 (46.7%)	

Outcome data

There was a significantly increased risk of both hospital length of stay of greater than one week (OR 3.38, 95% CI 1.31-8.72) and need for ICU admission (OR 3.18, 95% CI 1.26-8.05) in the high PCT group. Other primary and secondary outcomes, including ED readmission, in-hospital mortality, need for endotracheal intubation, and ICU length of stay greater than one week did not reach the level of significance (Table 2).

**Table 2:** Outcome Data * Denotes statistically significant difference between groups (p<0.05). OR: odds ratio, CI: confidence interval, LOS: length of stay, ICU: intensive care unit, ED: emergency department, N/A: not applicable, Trach: preexisting tracheostomy tube, <=: less than or equal to, >: greater than.

		Procalcitonin <=0.25ng/ml	Procalcitonin >0.25ng/ml	P value	Unadjusted OR (95% CI)
Total (n)		57 (65.5%)	30 (34.5%)	-	-
Hospital LOS	>7 days	13 (22.8%)	15 (50.0%)	*0.010	3.38 (1.31-8.72)
	<=7 days	44 (77.2%)	15 (50.0%)		
ICU admission	Yes	22 (38.6%)	20 (66.7%)	*0.013	3.18 (1.26-8.05)
	No	35 (61.4%)	10 (33.3%)		
ED readmission	Yes	16 (28.1%)	8 (26.7%)	1	1.00 (0.36-2.75)
	No	38 (66.6%)	19 (63.3%)		
	N/A (Deceased)	3 (5.3%)	3 (10%)		
Mortality	Yes	3 (5.3%)	3 (10.0%)	0.407	2.00 (0.38-10.58)
	No	54 (94.7%)	27 (90.0%)		
Intubation	Yes	6 (10.5%)	5 (16.7%)	0.311	1.93 (0.53-7.00)
	No	51 (89.5%)	22 (73.3%)		
	N/A (Trach)	0 (0%)	3 (10%)		
ICU LOS	>7 days	5 (8.8%)	5 (16.7%)	0.272	2.08 (0.55-7.85)
	<=7 days	52 (91.2%)	25 (83.3%)		

## Discussion

This study suggests that an increased PCT level in patients with COPD admitted to the hospital with respiratory illness is associated with increased healthcare resource utilization and more significant disease. Notably, patients with PCT levels greater than 0.25ng/ml are more likely to remain in the hospital for longer than one week or be admitted to the ICU during their hospital stay. It is likely that the increased resource utilization (as noted by increased ICU admission and longer length of stay) noted in the high PCT group is due to concomitant infectious etiology, either causing the exacerbation of COPD, or as the primary driver of respiratory failure. As noted, there was an increased incidence of lobar opacity on chest imaging in the high PCT group. Although, this did not necessarily lead to more antibiotic administration in that cohort. There was, however, a statistically significant decrease in corticosteroid utilization in the high PCT group, presumably due to the perceived notion that corticosteroids may worsen any possible infection.

This study analyzed the use of a relatively inexpensive and rapidly available test in all patients with a diagnosis of COPD and respiratory complaint. The measurement of PCT has most frequently been studied early in patients' hospital courses, whether it be to grade sepsis [12], evaluate for the cause of pneumonia [13], or to screen for bacterial meningitis [14]. Here we add to the extant data by showing that PCT also correlates with disease severity in acute exacerbations of COPD. This information could be used to develop new, or enhance existing, risk-stratification scores such as DECAF to help identify patients who may benefit from an increased level of care [15,16].

Limitations of this study include its performance at a single institution and small sample size overall. The dataset may have been limited due to the use of ICD-10 codes to identify patients and the lack of PFT data to adequately classify patients into baseline disease severity by GOLD criteria. Depending on how accurately the visit was coded, this may have led to some patients with COPD being omitted and the inclusion of patients with a clinical diagnosis of COPD who may not otherwise have obstructive spirometry, possibly limiting the external validity of the study. Lastly, over a third of all patients in this study had an elevated PCT above the upper limit of normal which perhaps limits its utility in risk-stratification scores due to being too prevalent of a risk factor.

## Conclusions

COPD continues to impose a significant burden on healthcare expenditure and hospital utilization. With improved prognostic testing, better resource management and patient care can be realized in COPD patients. PCT levels have a significant correlation with resource use during an acute hospitalization and could be used to help risk stratify patients being admitted to the hospital with COPD exacerbations. A prospective validation study, perhaps adding PCT to an existing severity score, would be of benefit.
